# Supplementary material for: Under-Dominance Constrains the Evolution of Negative Autoregulation in Diploids
Source: PLoS Comput Biol. 2013 Mar 21;9(3):e1002992. doi: 10.1371/journal.pcbi.1002992 (PMC3605092; doi:10.1371/journal.pcbi.1002992)
Supplement: Figure S1 — Steeper repression functions further limit the range of mutations that escape under-dominance. The x-axis shows the geometric mean of the binding strength across the set of resident alleles, in units of , and the y-axis shows the size of mutations to binding site strength, as described in the main text. In the gray region, mutations to one of the binding sites result in increased response time in the mutant compared to the resident alleles. In the white region mutations result in decreased response time in the mutant compared to the resident alleles; only mutations that fall within the white region can invade a population. Mutant invasibility is shown for Hill coefficients (left), and (right). Weak binding occurs when . Response times are calculated by numerically integrating Eq. 1 from zero protein concentration to 90% of the equilibrium. The optimal binding strength in these graphs is corresponding to a background transcription rate . (PDF) [file pcbi.1002992.s001.pdf]

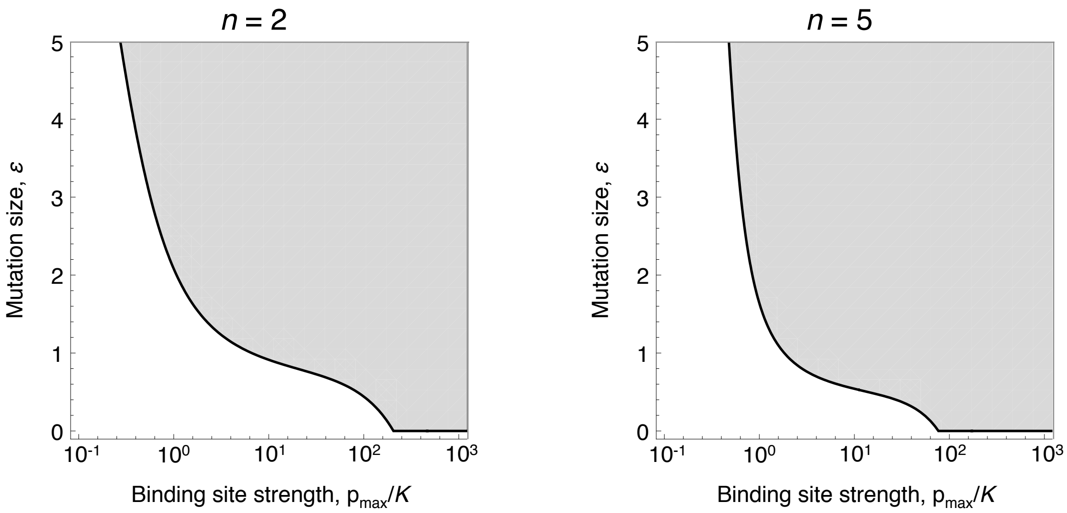

Figure S1: Steeper repression functions further limit the range of mutations that escape underdominance. The x-axis shows the geometric mean of the binding strength across the set of resident alleles, in units of p_{max}/K, and the y-axis shows the size of mutations to binding site strength, as described in the main text. In the gray region, mutations to one of the n binding sites result in increased response time in the mutant compared to the resident alleles. In the white region mutations result in decreased response time in the mutant compared to the resident alleles; only mutations that fall within the white region can invade a population. Mutant invasibility is shown for Hill coefficients $n = 2$ (left), and $n = 5$ (right). Weak binding occurs when $p_{max}/K \gtrsim 10^0$. Response times are calculated by numerically integrating Eq. 1 from zero protein concentration to 99% of the equilibrium. The optimal binding strength in these graphs is $p_{max}/K = 1250$ corresponding to a background transcription rate $k_l/k_0 = 10^{-3}$
