# Supplementary material for: Under-Dominance Constrains the Evolution of Negative Autoregulation in Diploids
Source: PLoS Comput Biol. 2013 Mar 21;9(3):e1002992. doi: 10.1371/journal.pcbi.1002992 (PMC3605092; doi:10.1371/journal.pcbi.1002992)
Supplement: Figure S2 — Increasing the Hill coefficient leads to slower response times unless binding strength is weak. The x-axis shows the binding strength in the resident allele, in units of , and the y-axis shows the ratio of response times for a heterozygote in which one allele has a Hill coefficient and the other has a Hill coefficient , to a homozygote with Hill coefficient . Below the gray dashed line, mutations result in increased response time in the mutant compared to the resident allele. Weak binding occurs when . Response times are calculated by numerically integrating Eq. 1 from zero protein concentration to 90% of the equilibrium. The optimal binding strength in these graphs is corresponding to a background transcription rate . (PDF) [file pcbi.1002992.s002.pdf]

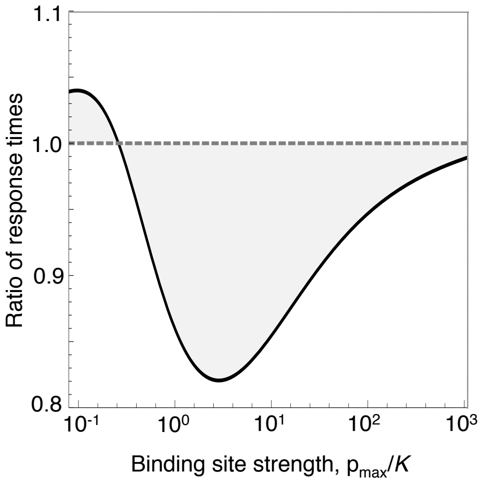

Figure S2: Increasing the Hill coefficient leads to slower response times unless binding strength is weak. The x-axis shows the binding strength in the resident allele, in units of p_{max}/K, and the y-axis shows the ratio of response times for a heterozygote in which one allele has a Hill coefficient $n = 1$ and the other has a Hill coefficient $n = 2$, to a homozygote with Hill coefficient $n = 1$. Below the gray dashed line, mutations result in increased response time in the mutant compared to the resident allele. Weak binding occurs when $p_{max}/K \gtrsim 10^0$. Response times are calculated by numerically integrating Eq. 1 from zero protein concentration to 99% of the equilibrium. The optimal binding strength in these graphs is $p_{max}/K = 1250$ corresponding to a background transcription rate $k_l/k_0 = 10^{-3}$
