# Supplementary material for: Under-Dominance Constrains the Evolution of Negative Autoregulation in Diploids
Source: PLoS Comput Biol. 2013 Mar 21;9(3):e1002992. doi: 10.1371/journal.pcbi.1002992 (PMC3605092; doi:10.1371/journal.pcbi.1002992)
Supplement: Figure S3 — Changing the background rate of transcription does not substantially alter the impact of under-dominance on mutations of size .The x-axis shows the binding strength in the resident allele, in units of , and the y-axis shows the size of mutations to binding site strength, as described in the main text. In the gray region, mutations result in increased response time in the mutant compared to the resident allele. In the white region mutations result in decreased response time in the mutant compared to the resident allele; only mutations that fall within the white region can invade a population. Mutant invasibility is shown for background transcription rates (left), and (right). Weak binding occurs when . Response times are calculated by numerically integrating Eq. 1 from zero protein concentration to 90% of the equilibrium. (PDF) [file pcbi.1002992.s003.pdf]

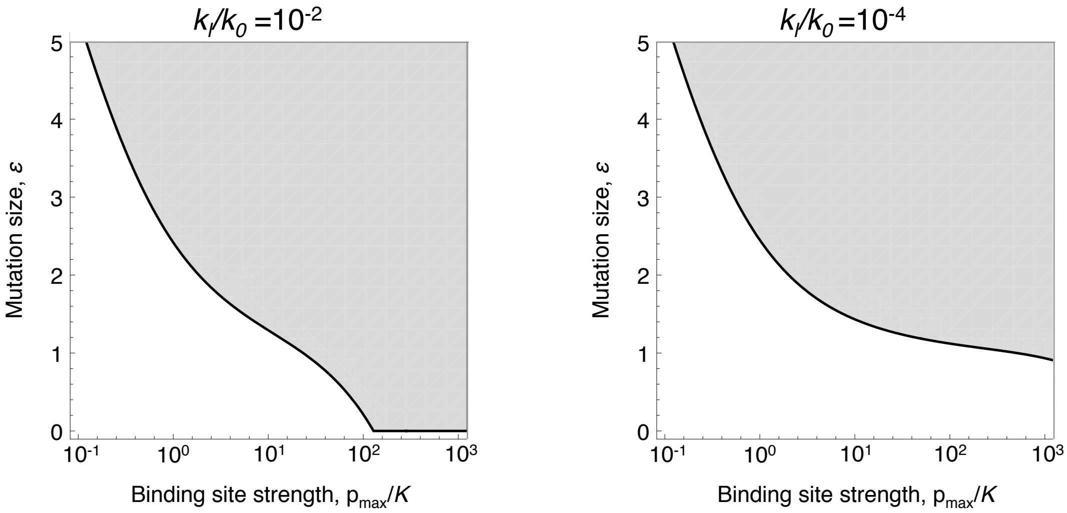

Figure S3: Changing the background rate of transcription does not substantially alter the impact of under-dominance on mutations of size $\epsilon > 2$.The x-axis shows the binding strength in the resident allele, in units of p_{max}/K, and the y-axis shows the size of mutations to binding site strength, as described in the main text. In the gray region, mutations result in increased response time in the mutant compared to the resident allele. In the white region mutations result in decreased response time in the mutant compared to the resident allele; only mutations that fall within the white region can invade a population. Mutant invasibility is shown for background transcription rates $k_l/k_0 = 10^{-2}$ (left), and $k_l/k_0 = 10^{-4}$ (right). Weak binding occurs when $p_{max}/K \gtrsim 10^0$. Response times are calculated by numerically integrating Eq. 1 from zero protein concentration to 99% of the equilibrium.
