# Supplementary material for: Under-Dominance Constrains the Evolution of Negative Autoregulation in Diploids
Source: PLoS Comput Biol. 2013 Mar 21;9(3):e1002992. doi: 10.1371/journal.pcbi.1002992 (PMC3605092; doi:10.1371/journal.pcbi.1002992)
Supplement: Figure S4 — Changing degradation rates changes response times but does not allow autoregulation to escape under-dominance. The figure shows results for the response time of autoregulating genes, to return to of their equilibrium. (left) Percentage change in the response time of a heterozygote compared to the resident homozygote. These are shown for different protein degradation rates coefficients, (black), (red) and (blue). Mutations become deleterious in the heterozygote when . (right) Percentage change in the response time of a mutant homozygote compared to the resident homozygote. Mutations become deleterious in the mutant homozygote when is about . The graphs show the results of stochastic simulations (see Materials and Methods) for parameter values typical for transcription factors, , , and . The resident homozygote has binding strength (as indicated by the x-axis), mutations are of size . (PDF) [file pcbi.1002992.s004.pdf]

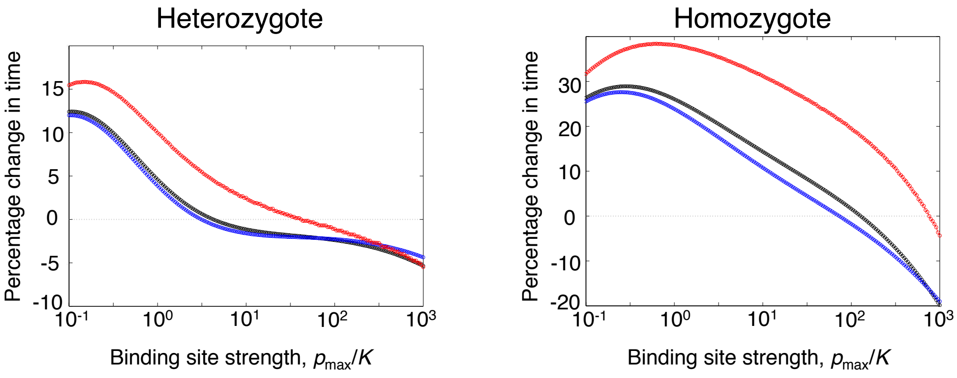

Figure S4: Changing degradation rates changes response times but does not allow autoregulation to escape under-dominance. The figure shows results for the response time of autoregulating genes, to return to 90% of their equilibrium. (left) Percentage change in the response time of a heterozygote compared to the resident homozygote. These are shown for different protein degradation rates coefficients, $\gamma_p = \frac{1}{3600}$ (black), $\gamma_p = \frac{1}{36000}$ (red) and $\gamma_p = \frac{1}{360}$ (blue). Mutations become deleterious in the heterozygote when $p_{max}/K > 1$. (right) Percentage change in the response time of a mutant homozygote compared to the resident homozygote. Mutations become deleterious in the mutant homozygote when p_{max}/K is about 10. The graphs show the results of stochastic simulations (see Materials and Methods) for parameter values typical for transcription factors, $k_r = 0.01s^{-1}$, $k_p = 0.17s^{-1}$, $k_l = 0.001s^{-1}$ and $\gamma_r = \frac{1}{120}s^{-1}$. The resident homozygote has binding strength p_{max}/K (as indicated by the x-axis), mutations are of size $\epsilon = 2$.
