# Supplementary material for: Under-Dominance Constrains the Evolution of Negative Autoregulation in Diploids
Source: PLoS Comput Biol. 2013 Mar 21;9(3):e1002992. doi: 10.1371/journal.pcbi.1002992 (PMC3605092; doi:10.1371/journal.pcbi.1002992)
Supplement: Figure S5 — Invasibility of autoregulatory binding sites. The response time of mutant (left) homozygotes and (right) heterozygotes are shown. Different values of the binding strength of the resident allele, in units of (x-axis), are plotted against mutations to binding site strength of different size (y-axis). Thus the graphs compare a resident allele, with a mutant allele, . Mutations falling into white region result in decreased response time in the carrier compared to resident genotype and are favoured by selection; mutations falling into the gray region result in increased response time and are not favoured by selection. Weak binding occurs when [1], [2]. Response times were calculated by numerically integrating Eq. 1 from zero protein concentration to 99% of the equilibrium. The optimal binding strength in these graphs is , corresponding to a background transcription rate . (PDF) [file pcbi.1002992.s005.pdf]

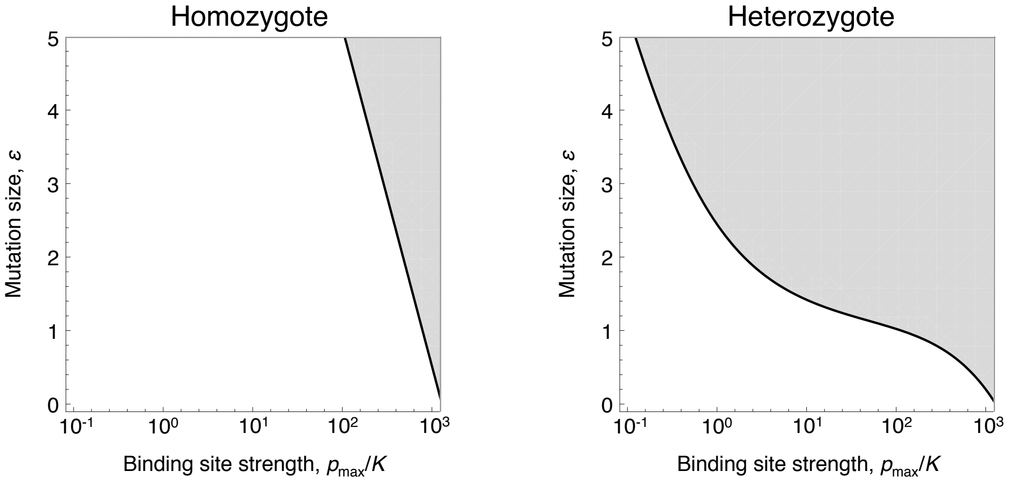

Figure S5: Invasibility of autoregulatory binding sites. The response time of mutant (left) homozygotes and (right) heterozygotes are shown. Different values of the binding strength of the resident allele, in units of p_{max}/K (x-axis), are plotted against mutations to binding site strength ϵ of different size (y-axis). Thus the graphs compare a resident allele, K_1 with a mutant allele, $K_2 = K_1 \exp[-\epsilon]$. Mutations falling into white region result in decreased response time in the carrier compared to resident genotype and are favoured by selection; mutations falling into the gray region result in increased response time and are not favoured by selection. Weak binding occurs when $p_{max}/K \gtrsim 10^0$ [10, 11]. Response times were calculated by numerically integrating Eq. 1 from zero protein concentration to 99% of the equilibrium. The optimal binding strength in these graphs is $p_{max}/K = 1250$, corresponding to a background transcription rate $k_l/\gamma_p = 10^{-3}$.
