# Supplementary material for: Under-Dominance Constrains the Evolution of Negative Autoregulation in Diploids
Source: PLoS Comput Biol. 2013 Mar 21;9(3):e1002992. doi: 10.1371/journal.pcbi.1002992 (PMC3605092; doi:10.1371/journal.pcbi.1002992)
Supplement: Table S1 — Autoregulation in Saccharomyces cerevisiae [10], [13], [14], [34]–[36]. (PDF) [file pcbi.1002992.s006.pdf]

**Gene**	**Autoregulation**	**Evidence**	**Function**	**Chromosome**
Pdr3	Positive	Direct	Activates the pleiotropic drug resistance network	II
Tec1	Positive (cooperative only)	Direct	Targets filamentation genes and *Ty1* expression	II
Smp1	Positive	General function	Putative transcription factor involved in regulating the response to osmotic stress	II
Nrg1	Negative	General function	Mediates glucose repression and negatively regulates a variety of processes including filamentous growth and alkaline pH response	IV
Yap6	Dual	General function	Basic leucine zipper (bZIP) transcription factor	IV
Sum1	Negative	General function	Required for mitotic repression of middle sporulation-specific genes	IV
Aro80	Positive	General function	Zinc finger transcriptional activator of the Zn2Cys6 family	IV
Swi4	Positive	General function	DNA binding component of the SBF complex (Swi4p-Swi6p)	V
Aft1	Positive	General function	Involved in iron utilization and homeostasis	VII
Sut1	Positive	General function	Involved in induction of hypoxic gene expression	VII
Ste12	Positive	General function	Activates genes involved in mating or pseudohyphal/invasive growth pathways	VIII
Stb5	Not specified	-	Involved in regulating multidrug resistance and oxidative stress response	VIII
Zap1	Positive	Direct	Binds to zinc-responsive promoters to induce transcription of certain genes in presence of zinc, represses other genes in low zinc	X
Hap1	Dual	General function	Involved in the complex regulation of gene expression in response to levels of heme and oxygen	XII
Arg81	Negative	General function	Involved in the regulation of arginine-responsive genes	XIII

Table S1: Autoregulation in *Saccharomyces cerevisiae* [4, 5, 6, 7, 8, 9].
