# Supplementary material for: Under-Dominance Constrains the Evolution of Negative Autoregulation in Diploids
Source: PLoS Comput Biol. 2013 Mar 21;9(3):e1002992. doi: 10.1371/journal.pcbi.1002992 (PMC3605092; doi:10.1371/journal.pcbi.1002992)
Supplement: Table S2 — Autoregulation in Humans [13]–[15], [35], [36]. (PDF) [file pcbi.1002992.s007.pdf]

Gene	Autoregulation	Evidence	Function	Chromosome
AP-2alphaA	Positive	General function	Controls keratinocyte-specific gene expression	-
Fos	Negative	Direct	Activator or repressor as a heterodimer with *Jun*	14
Jun	Positive	General function	Activator, in particular of genes required for DNA synthesis and cell proliferation	1
Myc	Negative	Direct	Causes release of cytochrome C from mitochondria and subsequent Caspase-9 processing	8
Elk1	Positive	General function	Mediates insulin-increased transcription of the prolactin gene in pituitary cells	X
Gli1	Positive	Direct	Gli-1 intracellular localization regulated by nucleocytoplasmic shuttling by Su(fu) protein	12
Nr3c1	Dual	General function	Activator or repressor in response to glucocorticoid hormones	5
Hnf4a	Positive	General function	Not specified	20
Nfkb2	Positive	General function	Activator when present as heterodimer with RelA	10
Irf1	Positive	General function	Positive regulator of interferon-beta and IFN-induced genes	5
Maz	Negative	Direct	Involved in transcriptional initiation and termination at the c-myc P2 promoter	16
Nfkb1	Positive	General function	Key regulator of genes involved in responses to infection, inflammation, stress	4
Tp53	Dual	Direct	Tumor suppressor, required for cells to pass the G1-S checkpoint	17
Sp1	Positive	Direct	Important regulator of keratinocyte-specific gene expression	12

Table S2: Autoregulation in Humans [2, 6, 7, 8, 9] (continued below).

**Gene**	**Autoregulation**	**Evidence**	**Function**	**Chromosome**
Srf	Dual	General function	Induces an optimal TFIID conformation for recruitment of the transcription preinitiation complex	6
Sry	Not specified	-	Sex-determining region Y gene product; testis-determining factor	Y
Runx1	Positive	General function	Related to t(8,21) acute myeloid leukemia	21
Atf3	Negative	Direct	Plays a critical role in accelerating caspase protease activation and apoptosis	1
Fosl1	Positive	Direct	Activator as a heterodimer with Jun factors	11
Stat1	Positive	General function	Acts synergistically with NF-kappaB for activation of inflammatory genes	2
*E2f*1	Positive	Direct	Regulates the expression of Apaf-1, the gene for apoptosis protease-activating factor 1	-
Spi1	Positive	General function	Binding sites cooperate for activation of some myeloid-specific promoters	11
Sp3	Negative	General function	Expression of Sp1 and Sp3 was found co-regulated in fibrosarcoma cell lines	2
Phox2b	Positive	General function	Activates gene expression of DBH and c-Fos T00123 in response to both the PKA and PKC	4
Irf7	Not specified	-	Not specified	11
Irf4	Positive	General function	essential for function of mature B- and T-lymphocytes	-
Tp73	Not specified	-	Not specified	1
Tp63	Not specified	-	Not specified	3

Table S2: Autoregulation in Humans continued [2, 6, 7, 8, 9].
