# Supplementary material for: Under-Dominance Constrains the Evolution of Negative Autoregulation in Diploids
Source: PLoS Comput Biol. 2013 Mar 21;9(3):e1002992. doi: 10.1371/journal.pcbi.1002992 (PMC3605092; doi:10.1371/journal.pcbi.1002992)
Supplement: Table S3 — Autoregulation in Drosophila [12]–[14], [35], [36]. (PDF) [file pcbi.1002992.s008.pdf]

**Gene**	**Autoregulation**	**Evidence**	**Function**	**Chromosome**
Antp	Positive	Direct	Homeotic selector gene	3R
Cf2-II	Positive	General function	Late activator in follicle cells during chorion formation	-
Dfd	Positive	Direct	Homeotic selector gene product	3R
E(spl)	Negative	General function	Involved in lateral inhibition of AS-C proteins within neural clusters and in singling out one mother sensory cell per cell cluster	3R
Eip74EF	Positive	General function	Not specified	3L
Trl	Positive	General function	Disrupts nucleosomal structures leading to a rearrangement of nucleosomes through an energy-requiring process	3L
Ubx	Positive	Direct	Facilitates assembly of transcription initiation complex	3R
bab1	Negative	General function	Not specified	3L
ems	Positive	General function	Functions as a gap gene product in the first phase of its expression when it is expressed in a single anterior band where the head is developing	3R
en	Positive	Direct	Segment polarity gene	2R
eve	Positive	Direct	Pair-rule gene product, generally acting as repressor	2R
ftz	Positive	Direct	Pair-rule gene product, mutations of which may cause loss of half of larval segments	3R
gl	Positive	General function	Required for normal photoreceptor cell development	3R
gsb	Positive	General function	Segment polarity gene product specifying the identity of row 5 neuroblasts	2R

Table S3: Autoregulation in *Drosophila* [3, 6, 7, 8, 9] (continued below).

**Gene**	**Autoregulation**	**Evidence**	**Function**	**Chromosome**
hb	Positive	General function	Gap gene	3R
ovo	Negative	General function	Regulates female germ line development, maintenance and gametogenesis	X
slbo	Positive	General function	May be required for expression of gene products that mediate border cell migration	2R
tin	Dual	General function	Required for the development of dorsal mesoderm, including heart	3R
vvl	Positive	General function	Not specified	3L

Table S3: Autoregulation in *Drosophila* continued [3, 6, 7, 8, 9].
