# Supplementary material for: Under-Dominance Constrains the Evolution of Negative Autoregulation in Diploids
Source: PLoS Comput Biol. 2013 Mar 21;9(3):e1002992. doi: 10.1371/journal.pcbi.1002992 (PMC3605092; doi:10.1371/journal.pcbi.1002992)
Supplement: Text S1 — The supporting information text describes the methods used in constructing Tables S1, S2, S3 from curated databases, and in creating Fig. S1, S2, S3, S4, S5 by relaxing the assumptions of the model outlined in the main text. (PDF) [file pcbi.1002992.s009.pdf]

Supplementary information

Alexander J. Stewart, Andrew Pomiankowski, Robert M. Seymour and Max Reuter

Negatively autoregulating genes in yeast, *Drosophila* and humans

Below we list the known instances of autoregulating genes in yeast (Table S1), *Drosophila* (Table S2) and humans (Table S3), their functions, any documented co-regulatory interactions and the chromosome the gene is found on. Autoregulating genes are determined from systematic datasets on the regulatory interactions of the individual species [1, 2, 3, 4, 5, 6] and autoregulation is considered to be negative (or positive) on the basis of direct evidence or if a transcription factor is known to have a predominantly repressing (or activating) regulatory function based on its entry in the TRANSFAC database [6]. Where transcription factors are known to have both an activating and a repressing regulatory function, and no direct evidence on the type of autoregulation is available, the gene is listed as having "Dual" autoregulation. Dual autoregulation accounts for 2 instances in *S. cerevisiae*, 1 instance in *Drosophila* and 3 instances in humans. Where it is not known (on the basis of TRANSFAC) whether a transcription factor has a repressing or activating regulatory function, the type of autoregulation is listed as "Not specified". Such unspecified autoregulation accounts for 1 instance in *S. cerevisiae*, 6 instances in *Drosophila* and 4 instances in humans.

The impact of variation in other parameters on the evolution of autoregulatory binding sites

Our study of the evolution of autoregulating transcription factor binding sites focuses on the evolution of binding site strength, determined by the dissociation constant K. However our model includes a number of other parameters, which we estimate on the basis of empirical data in our simulations and in the numerical analysis of our model (Figs. 2-4, main text). In the following sections we investigate the impact on these results of varying the slope of the autoregulatory repression function, $\phi(p)$, the background rate of transcription, k_l and the rate of mRNA and protein degradation γ_r and γ_p.

Varying the slope of the repression function

The repression function $\phi(p)$ given in Eqs. 1 has a Michaelis-Menten-like form, which describes a single binding site. A more general form of $\phi(p)$ is given by a Hill function

$$\phi(p) = \frac{k_0}{1 + \left(\frac{p}{K}\right)^n}$$

where higher values of n correspond to a repression function with a steeper slope. Such a function corresponds to the case in which TFs bind cooperatively to n different binding sites, with K interpreted as the geometric mean of the individual binding strengths across the sites. To test the generality of our results in the main text we look at the invasibility of mutations that increase binding strength (decrease K) for values of $n > 1$ (i.e for repression functions with steeper slopes). The results are shown in Fig. S1. These show that increasing the slope of $\phi(p)$ tends to decrease

the range of available mutations to K that escape the effects of under-dominance, compared to the case where $n = 1$ (Fig. 2, main text). Therefore we conclude that autoregulation which is described by a steeper repression function cannot escape the effects of under-dominance.

We also investigate whether mutations that increase the Hill coefficient give rise to faster response times. Increasing the Hill coefficient may be interpreted as adding another autoregulatory binding site in the presence of cooperativity. Remarkably we find a similar pattern to that observed for the dissociation constant (Fig. S2): when binding strength is weak, mutations that increase n lead to faster response times, but when binding strength is strong, mutations that increase n tend to lead to slower response times. Thus mutations to n do not provide a way to circumvent the effects of under-dominance.

Variation in the background rate of transcription

Variation in the rate of background transcription, k_l changes the optimal binding site strength for which response time is fastest (see Appendix). In order to investigate the effects of variation in the background rate of transcription on the evolution of stronger autoregulation, we varied k_l by three orders of magnitude. Fig. 2b of the main text shows the invasibility of mutants in diploids for $k_l/k_0 = 10^{-3}$. Fig. S3 shows that variation in k_l by an order of magnitude above or below this value has little effect on the invasibility of mutants with $\epsilon > 1$, but can substantially alter the invasibility of mutants with small effect ($\epsilon < 1$), due to the position of the optimum value K_{opt} for which response time is fastest being varied.

Variation in protein and mRNA degradation rates

Variation in protein degradation rates offer an alternative method to negative autoregulation for decreasing the response time of genes to perturbation. Indeed, regulated degradation is used widely throughout eukaryotes for his purpose [7]. To determine whether variation in the rates of mRNA or protein degradation alter the ability of diploids to evolve negative autoregulataion for this purpose, we ran molecular simulations for a range of degradation rates. The results are shown in Fig. S4. We have plotted the percentage change in the response time for mutant heterozygotes and mutant homozygotes, using different protein degradation rates, γ_p^*. Fig. S4 shows that although increasing or decreasing protein degradation rates lead to faster or slower response times, they do not prevent mutations that increase the strength of negative autoregulation being deleterious. Thus variation in protein degradation rates do not offer a way for autoregulatory binding sites to escape the effects of under-dominance.

Changing the definition of response time

We have defined response time as the time to return to 90% of equilibrium expression. Clearly we could have chosen a different cut-off. To show that this does not alter our results Fig. S5 reproduces Fig. 2 with response time defined as the time to regurn to 99% of equilibrium expression.

References

[1] Bauer T, Eils R, König R (2011) Rip: the regulatory interaction predictor–a machine learning-based approach for predicting target genes of transcription factors. Bioinformatics 27: 2239-47.

[2] Bergman CM, Carlson JW, Celniker SE (2005) Drosophila dnase i footprint database: a systematic genome annotation of transcription factor binding sites in the fruitfly, drosophila melanogaster. Bioinformatics 21: 1747-9.

[3] Cherry JM, Ball C, Weng S, Juvik G, Schmidt R, et al. (1997) Genetic and physical maps of saccharomyces cerevisiae. Nature 387: 67-73.

[4] Lee TI, Rinaldi NJ, Robert F, Odom DT, Bar-Joseph Z, et al. (2002) Transcriptional regulatory networks in saccharomyces cerevisiae. Science 298: 799-804.

[5] Matys V, Kel-Margoulis OV, Fricke E, Liebich I, Land S, et al. (2006) Transfac and its module transcompel: transcriptional gene regulation in eukaryotes. Nucleic Acids Res 34: D108-10.

[6] Wingender E, Dietze P, Karas H, Knüppel R (1996) Transfac: a database on transcription factors and their dna binding sites. Nucleic Acids Res 24: 238-41.

[7] Raj A, van Oudenaarden A (2008) Nature, nurture, or chance: stochastic gene expression and its consequences. Cell 135: 216-226.

[8] Kersey PJ, Lawson D, Birney E, Derwent PS, Haimel M, et al. (2010) Ensembl genomes: extending ensembl across the taxonomic space. Nucleic Acids Res 38: D563-9.

[9] Kinsella RJ, Kähäri A, Haider S, Zamora J, Proctor G, et al. (2011) Ensembl biomarts: a hub for data retrieval across taxonomic space. Database (Oxford) 2011: bar030.

[10] Becskei A, Serrano L (2000) Engineering stability in gene networks by autoregulation. Nature 405: 590-3.

[11] Rosenfeld N, Elowitz MB, Alon U (2002) Negative autoregulation speeds the response times of transcription networks. J Mol Biol 323: 785-93.
